# Shexiang Baoxin Pill for Acute Myocardial Infarction: Clinical Evidence and Molecular Mechanism of Antioxidative Stress

**DOI:** 10.1155/2021/7644648

**Published:** 2021-11-30

**Authors:** Jianbo Guo, Zongshi Qin, Qingyong He, Tung Leong Fong, Ngai Chung Lau, William C. S. Cho, Hui Zhang, Peipei Meng, Xiaoxiao Xing, Min Li, Zhang-Jin Zhang, Haiyong Chen

**Affiliations:** ^1^School of Chinese Medicine, LKS Faculty of Medicine, The University of Hong Kong, Hong Kong, China; ^2^Guang'an Men Hospital, China Academy of Chinese Medical Sciences, Beijing, China; ^3^Department of Clinical Oncology, Queen Elizabeth Hospital, Hong Kong, China; ^4^Henan University of Chinese Medicine, Henan, China; ^5^Department of Chinese Medicine, The University of Hong Kong-Shenzhen Hospital, Shenzhen, China

## Abstract

Acute myocardial infarction (AMI) has been a preclinical and clinical concern due to high hospitalization rate and mortality. This study was aimed at evaluating the effectiveness and safety of Shexiang Baoxin Pill (SBP) for AMI and exploring the possible mechanism of oxidative stress. Six databases were searched on March 26, 2021. Twenty-four studies were included and accessed by the RoB 2.0 or SYRCLE tool. Compared with routine treatment (RT), SBP showed the effectiveness in the clinical efficacy (RR = 1.15, 95% CI [1.06, 1.25]), left ventricular ejection fraction (LVEF) (SMD = 0.73, 95% CI [0.62, 0.95]), glutathione (GSH) (SMD = 2.07, 95% CI [1.51, 2.64]), superoxide dismutase (SOD) (SMD = 0.92, 95% CI [0.58, 1.26]), malondialdehyde (MDA) (SMD = −4.23, 95% CI [-5.80, -2.66]), creatine kinase-myocardial band (CK-MB) (SMD = −4.98, 95% CI [-5.64, -4.33]), cardiac troponin I (cTnI) (SMD = −2.17, 95% CI [-2.57, -1.76]), high-sensitivity C-reactive protein (Hs-CRP) (SMD = −1.34, 95% CI [-1.56, -1.12]), interleukin-6 (IL-6) (SMD = −0.99, 95% CI [-1.26, -0.71]), triglycerides (TG) (SMD = −0.52, 95% CI [-0.83, -0.22]), flow-mediated dilation (FMD) (SMD = 1.39, 95% CI [1.06, 1.72]), von Willebrand Factor (vWF) (SMD = −1.77, 95% CI [-2.39, -1.15]), nitric oxide (NO) (SMD = 0.89, 95% CI [0.65, 1.13]), and recurrent rate (RR = 0.30, 95% CI [0.15, 0.59]). But SBP adjunctive to RT plus PCI had no improvements in almost pooled outcomes except for the Hs-CRP (SMD = −1.19, 95% CI [-1.44, -0.94]) and TG (SMD = −0.25, 95% CI [-0.48, -0.02]). Laboratory findings showed that SBP enhanced the endothelial nitric oxide synthase (eNOS) activity and regulated laboratory indexes especially for homocysteine. In conclusion, SBP has adjunctive effects on AMI via the mechanism of antioxidative stress. The current evidence supports the use of SBP for mild and moderate AMI patients.

## 1. Introduction

Acute myocardial infarction (AMI) is the sudden damage to the myocardium due to insufficient blood flow to the heart. It is characterized by chest pain, chest discomfort, and acute shortness of breath [1]. Globally, AMI has become the leading cause of hospitalization and death [2]. Early revascularization and primary percutaneous coronary intervention (PCI) restore blood flow to the culprit coronary artery and reduce AMI mortality rate [3, 4]. However, immediate multivessel PCI might cause additional risks, e.g., induction of further ischemia, volume overload, and renal impairment due to the use of an increased dose of contrast material [5, 6]. Concurrently, the abrupt restoration therapy of coronary flow may induce reversible impairment of myocardial contractility, ventricular arrhythmias, and microvascular dysfunction. The myocardial ischemia/reperfusion (I/R) injury leads to myocyte necrosis, slows cardiomyocyte healing, and results in heart failure [7, 8]. Thus, prevention of I/R injury in AMI could reduce the injury.

Shexiang Baoxin Pill (SBP) is a classical Chinese medicine (CM) formula for cardiovascular diseases including AMI and stable angina pectoris [9–11], which has been approved by the Chinese Food and Drug Administration [12]. A pharmacological study indicates that SBP reduces cardiac infarct volume, suppresses inflammation, and promotes angiogenesis in the heart [12]. SBP is composed of 7 Chinese medicines or extracts including Moschus, Radix Ginseng, Calculus Bovis, Cortex Cinnamomi, Styrax, Venenum Bufonis, and Borneolum Syntheticum. Ginsenosides and cinnamaldehyde, active components of SBP, regulate energy metabolism in cardiomyocytes [13] and inhibit reactive oxygen species (ROS) production and autophagy [14]. However, the efficacy and mechanisms of SBP for AMI have not been systematically evaluated. This review was aimed at evaluating the efficacy and mechanisms of SBP through clinical studies and experimental studies with AMI animal models.

## 2. Materials and Methods

### 2.1. Search Strategy

Following the Preferred Reporting Items for Systematic Reviews and Meta-Analyses (PRISMA) statement (Supplementary Table 1) [15], this registered study (PROSPERO, no. CRD42021245957) searched six electronic databases: PubMed, Embase, Cochrane, Chinese National Knowledge Infrastructure (CNKI), China Science and Technology Journal (VIP), and Wanfang, from the date of database establishment to March 26, 2021. The combination of MeSH terms and keywords were used as follows: “Shexiang Baoxin” AND “acute myocardial infarction”. The PubMed database retrieval strategy is shown in Supplementary Table 2.

### 2.2. Eligibility Criteria

Inclusion criteria were as follows: (1) preclinical experiment (PE) or randomized controlled trial (RCT) studying SPB; (2) animal models of AMI were induced by operation ligation [16, 17], or patients met diagnostic criteria for AMI [18–20]; (3) SBP was an explored mechanism in preclinical experiments and was used as intervention or adjunctive to routine treatment (RT) in the clinical observation group; and (4) the clinical observation group and control group received RT or PCI. Exclusion criteria were as follows: (1) repetitive studies, comment, clinical experience, case report, review, data mining research, and protocol; (2) non-RCT and PE not studying oxidative stress; (3) PE or RCT contained the intervention of moxibustion, acupuncture, or other CM except for SBP; and (4) the study lacked essential data even though the principal authors were contacted.

### 2.3. Data Extraction

(1) Basic information of the included experiments (the first author, publication year, animal species, sex, number of animals, weight, intervention, and experiment duration) and trials (the first author, publication year, sample size, age information of the patients, intervention, and trial duration) were extracted; (2) all outcome indicators of experiments were extracted; (3) the primary outcome indicator (clinical efficacy rate) and second outcome indicators (cardiac function, oxidative stress, myocardial enzyme, inflammatory cytokines, blood lipid level, vascular endothelial function, and complication rate) of trials were extracted; and (4) endpoint data and baseline data were extracted for each outcome.

### 2.4. Quality Assessment

Six aspects of the version 2 of the Cochrane Risk of Bias Tool (RoB 2.0) [21] were assessed for the included RCTs: randomization process, deviations from the intended interventions, missing outcome data, outcome measurements, selection of the reported results, and overall bias according to the three criteria of “low risk,” “high risk,” or “some concerns.” Two researchers (JG and ZQ) assessed the included studies individually, and the third researcher (HC) resolved the discrepancies. GRADE (Grading of Recommendations, Assessment, Development and Evaluations) was used to evaluate evidence certainty of meta-analysis results. The Systematic Review Centre for Laboratory Animal Experimentation (SYRCLE) [22] risk of bias tool was used for Pes, including sequence generation, baseline characteristics, allocation concealment, random housing, blinding of performance, random outcome assessment, blinding of detection, incomplete outcome data, selective outcome reporting, and other sources of bias.

### 2.5. Statistical Analysis

The Stata 17.0 software (Stata Corp., College Station, TX, USA) was applied to statistical analysis: (1) a random effects model was adopted for pooling studies with high heterogeneity while a fixed effects model was applied for studies with low heterogeneity; (2) Cohen's *d* and 95% CI were used for continuous variables; (3) RR (relative risk) and 95% CI were used for categorical variables; (4) weight (%) was used to indicate a percentage of each study contributing to the pooled intervention effects; (5) a *p* value < 0.05 was considered statistically different; (6) heterogeneity was evaluated by *Q* statistics and *I*^2^, and the *p* value in *Q* statistics was <0.05 or *I*^2^ > 50% presented high heterogeneity or otherwise low heterogeneity; and (7) sensitivity analysis and subgroup analysis were carried out when studies had significant heterogeneity.

## 3. Results

### 3.1. Eligible Studies

**Figure 1:**
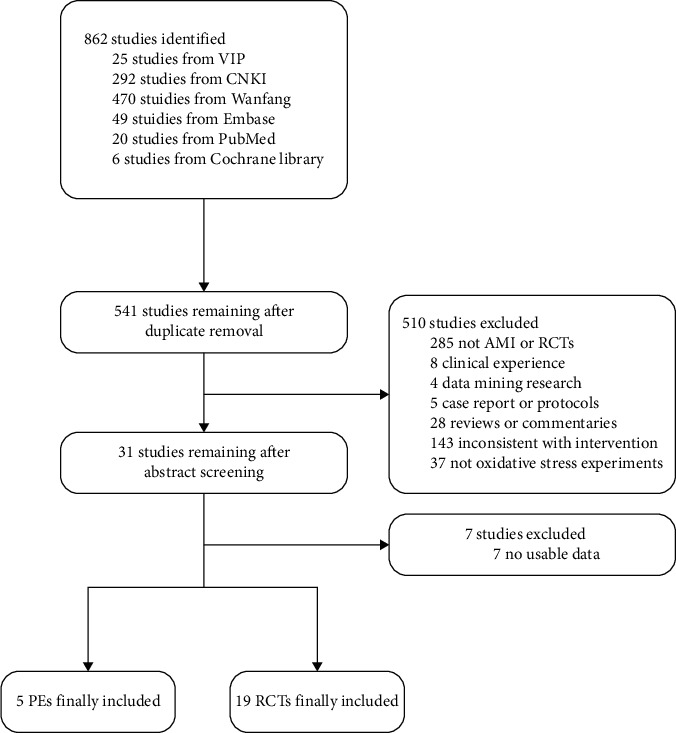
Flow chart.

A total of 862 studies were retrieved through the initial search, and 541 studies were obtained after screening out duplicate studies. Subsequently, 510 studies were excluded by reviewing titles or abstracts. In the remaining 31 studies, 7 studies were excluded following the full-text screen. The flow chart of screening is shown in Figure 1.

### 3.2. Study Characteristics

Nineteen RCTs [23–41] and five PEs [42–46] were included in this study, involving 1849 patients (observation group: 926, control group: 923) and 209 animals, respectively. The years of publication range from 1999 to 2021. The shortest experimental duration is 5 days, and the longest is 15 days. As for RCTs, the shortest duration of treatment is 2 weeks, and the longest is 24 weeks. The number of animals ranges from 6 to 81, and the sample size of patients is from 59 to 200 in each study. In the included PEs, three experiments [43–45] were carried out in metabolomics, one experiment [42] involved pharmacodynamics, and one experiment [46] was carried out in quantitative proteomics. In the included RCTs, 5 trials [37–41] used PCI both in the observation group and in the control group. The basic information of included studies is shown in Tables 1 and 2.

### 3.3. Risk of Bias

Five PEs [42–46] were assessed by the SYRCLE risk of bias tool (Supplementary Table 3). Due to lack of reporting, random housing and blinding of performance were judged as “some concerns” in all PEs. Three PEs [42, 44, 45] without reporting randomization were judged as “some concerns,” and three PEs neglected specific baseline report and thus increased the risk of bias, which were judged as “some concerns.” Nineteen RCTs [23–41] were assessed by using the RoB 2.0 tool (Supplementary Table 4). Among the assessments, four RCTs [27–29, 37] with a good design and report were assessed as “low” risks, but almost RCTs presented the risk of “some concerns” due to missing outcome data or selection of the reported result; even two RCTs [34, 36] with incomplete data or results led to the “high” risks.

### 3.4. Preclinical Experiments

Five experiments [42–46] were included that studied SBP for AMI in AMI animal models. The histology analysis in an experiment [42] showed that the endothelial nitric oxide synthase (eNOS) was mainly distributed in the myocardial interstitium, and SBP increased the expression of eNOS in the left ventricle. The metabonomics analysis showed that SBP downregulated hippuric acid, homocysteine, 5-methylcytosine, PGPC, and allantoin, which were involved in oxidative injury [43]. Another study analyzed the effects of SBP ginsenosides and found that ginsenoside Rg1 and Rb3 downregulated indoleacrylic acid, Rc upregulated 6-hydroxymelatonin and downregulated thymidine, and Re downregulated thymidine, indicating antioxidative effects of SBP [44]. SBP downregulated homocysteine to protect against ROS-induced endothelial cell injury [43, 45]. The proteomics analysis showed that peroxiredoxin-3 in SBP protects against oxidative stress in a rat model of myocardial infarction [46].

### 3.5. Clinical Trials

#### 3.5.1. Clinical Efficacy

**Figure 2:**
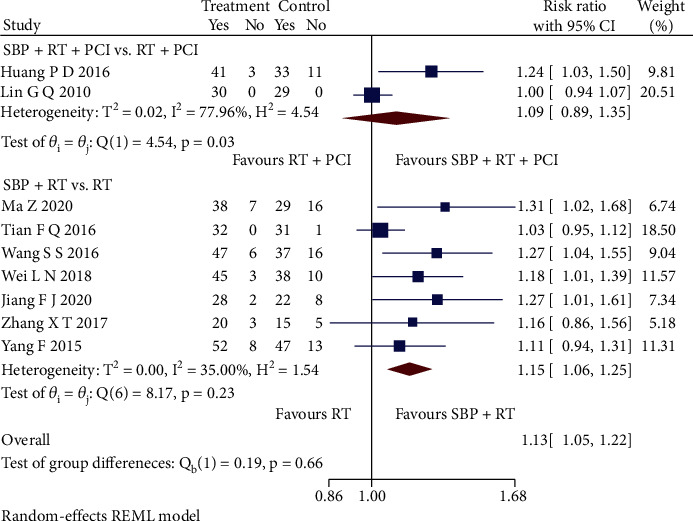
Forest plot of subgroup analysis on the clinical efficacy rate.

Nine RCTs [27–29, 31–33, 35, 37, 40] reporting a clinical efficacy rate were pooled. Among the subgroup analysis (Figure 2), SBP plus RT showed a better clinical efficacy rate than RT alone (RR = 1.15, 95% CI [1.06, 1.25], *p* < 0.05) with low heterogeneity (*Q* (6) = 8.17, *p* = 0.23, *I*^2^ = 35.00%). But SBP adjunctive to RT plus PCI did not significantly improve the clinical efficacy rate (RR = 1.09, 95% CI [0.89, 1.35], *p* > 0.05) and showed high heterogeneity (*Q* (1) = 4.54, *p* = 0.03, *I*^2^ = 77.96%).

#### 3.5.2. Cardiac Function

**Figure 3:**
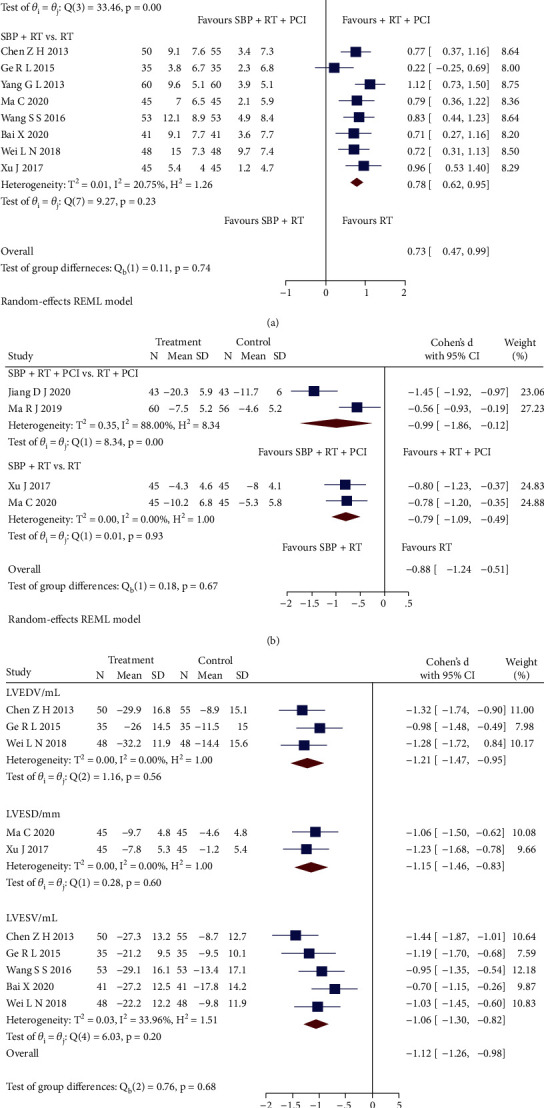
(a) Forest plot of subgroup analysis on the LVEF. (b) Forest plot of subgroup analysis on the LVEDD. (c) Forest plot of subgroup analysis on the LVEDV, LVESD, and LVESV.

Twelve RCTs [23, 25–27, 29–31, 34, 37, 38, 40, 41] reporting cardiac function were pooled and conducted subgroup analysis, as shown in Figures 3(a)–3(c). The subgroup analysis revealed that SBP plus RT significantly presented a higher left ventricular ejection fraction (LVEF) level than RT (SMD = 0.73, 95% CI [0.62, 0.95], *p* < 0.05; *Q* (7) = 9.27, *p* = 0.23, *I*^2^ = 20.75%, low heterogeneity), but SBP adjunctive to RT plus PCI had no significant difference (SMD = 0.64, 95% CI [-0.16, 1.45], *p* > 0.05; *Q* (3) = 33.46, *p* < 0.05, *I*^2^ = 92.31%, high heterogeneity) compared with RT plus PCI. Sensitivity analysis indicated that differences in PCI operations could cause the heterogeneity. Further, compared with RT, SBP plus RT had a significant improvement in left ventricular end-diastolic diameter (LVEDD) (SMD = −0.79, 95% CI [-1.09, -0.49], *p* < 0.05; *Q* (1) = 0.01, *p* = 0.93, *I*^2^ = 0, low heterogeneity), left ventricular end-diastolic volume (LVEDV) (SMD = −1.21, 95% CI [-1.47, -0.95], *p* < 0.05; *Q* (2) = 1.16, *p* = 0.56, *I*^2^ = 0, low heterogeneity), left ventricular end-systolic diameter (LVESD) (SMD = −1.15, 95% CI [-1.46, -0.83], *p* < 0.05; *Q* (1) = 0.28, *p* = 0.60, *I*^2^ = 0, low heterogeneity), and left ventricular end-systolic volume (LVESV) (SMD = −1.06, 95% CI [-1.30, -0.82], *p* < 0.05; *Q* (4) = 6.03, *p* = 0.20, *I*^2^ = 33.96%, low heterogeneity). Besides, SBP adjunctive to RT plus PCI had a significant improvement in LVEDD (SMD = −0.99, 95% CI [-1.86, -0.12], *p* < 0.05) with the high heterogeneity (*Q* (1) = 8.34, *p* < 0.05, *I*^2^ = 88.00%). Sensitivity analysis indicated that the heterogeneity could be caused by measuring methods.

#### 3.5.3. Oxidative Stress

**Figure 4:**
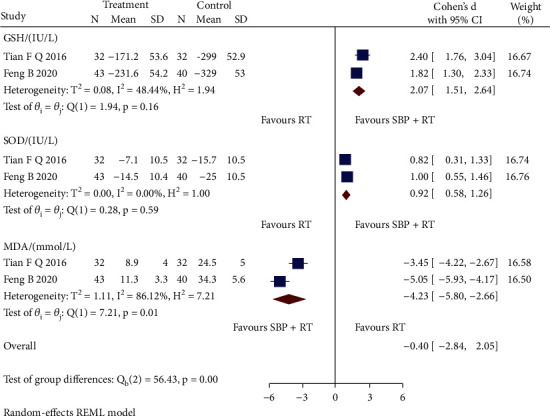
Forest plot of subgroup analysis on the GSH, SOD, and MDA.

Based on the reports of three oxidative stress indicators, two RCTs [28, 36] were pooled, as shown in Figure 4. Compared with RT, SBP adjunctive to RT had significant effectiveness of increasing both glutathione (GSH) level (SMD = 2.07, 95% CI [1.51, 2.64], *p* < 0.05; *Q* (1) = 1.94, *p* = 0.16, *I*^2^ = 48.44%, moderate heterogeneity) and superoxide dismutase (SOD) level (SMD = 0.92, 95% CI [0.58, 1.26], *p* < 0.05; *Q* (1) = 0.28, *p* = 0.59, *I*^2^ = 0, low heterogeneity) and decreasing malondialdehyde (MDA) level (SMD = −4.23, 95% CI [-5.80, -2.66], *p* < 0.05; *Q* (1) = 7.21, *p* = 0.01, *I*^2^ = 86.12%, high heterogeneity). Sensitivity analysis indicated that differences in measuring time could cause the heterogeneity.

#### 3.5.4. Laboratory Indexes

**Figure 5:**
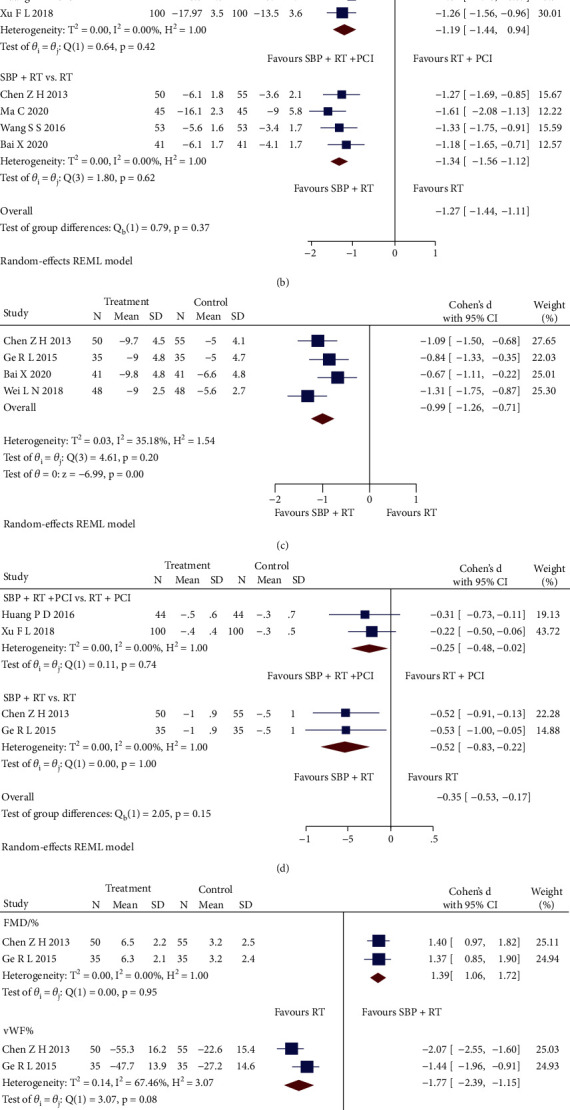
(a) Forest plot of subgroup analysis on the CK-MB and cTnI. (b) Forest plot of subgroup analysis on the Hs-CRP. (c) Forest plot of pooled analysis on the IL-6. (d) Forest plot of subgroup analysis on the TG. (e) Forest plot of subgroup analysis on the FMD and vMF. (f) Forest plot of pooled analysis on the NO.

*(1) Myocardial Enzymes*. Two RCTs [28, 36] reported creatine kinase-myocardial band (CK-MB) and cardiac troponin I (cTnI). The subgroup analysis showed that SBP plus RT had significant effectiveness in CK-MB (SMD = −4.98, 95% CI [-5.64, -4.33], *p* < 0.05; *Q* (1) < 0.05, *p* = 1.00, *I*^2^ = 0, low heterogeneity) and cTnI (SMD = −2.17, 95% CI [-2.57, -1.76], *p* < 0.05; *Q* (1) = 0.01, *p* = 0.94, *I*^2^ = 0, low heterogeneity) (Figure 5(a)).

*(2) Inflammatory Factors*. SBP plus RT significantly declined the levels of high-sensitivity C-reactive protein (Hs-CRP) (SMD = −1.34, 95% CI [-1.56, -1.12], *p* < 0.05; *Q* (3) = 1.80, *p* = 0.62, *I*^2^ = 0, low heterogeneity) and interleukin-6 (IL-6) (SMD = −0.99, 95% CI [-1.26, -0.71], *p* < 0.05; *Q* (3) = 4.61, *p* = 0.20, *I*^2^ = 35.18%, low heterogeneity), as shown in the pooled analysis of six RCTs [23, 25, 27, 29–31] (Figures 5(b)and 5(c), respectively). The pooled analysis of other two RCTs [37, 39] showed that SBP adjunctive to RT plus PCI had a significant improvement in Hs-CRP (SMD = −1.19, 95% CI [-1.44, -0.94], *p* < 0.05; *Q* (1) = 0.64, *p* = 0.42, *I*^2^ = 0, low heterogeneity) (Figure 5(b)).

*(3) Blood Lipid Level*. Four RCTs reporting triglyceride (TG) level were pooled by subgroup analysis of interventions. As shown in Figure 5(d), both SBP plus RT (SMD = −0.52, 95% CI [-0.83, -0.22], *p* < 0.05; *Q* (1) < 0.05, *p* = 1.00, *I*^2^ = 0, low heterogeneity) and SBP adjunctive to RT plus PCI (SMD = −0.25, 95% CI [-0.48, -0.02], *p* < 0.05; *Q* (1) = 0.11, *p* = 0.74, *I*^2^ = 0, low heterogeneity) significantly lowered TG level when compared with RT and RT plus PCI, respectively.

*(4) Vascular Endothelial Function*. Compared with RT, SBP plus RT had significant improvements in the flow-mediated dilation (FMD) level (SMD = 1.39, 95% CI [1.06, 1.72], *p* < 0.05; *Q* (1) < 0.05, *p* = 0.95, *I*^2^ = 0, low heterogeneity) and von Willebrand Factor (vWF) level (SMD = −1.77, 95% CI [-2.39, -1.15], *p* < 0.05; *Q* (1) = 3.07, *p* = 0.08, *I*^2^ = 67.46%, high heterogeneity) in the subgroup analysis of two RCTs [23, 25] (Figure 5(e)). Sensitivity analysis indicated that differences in the measuring method could contribute to the heterogeneity. Three RCTs [27, 29, 31] reporting the nitric oxide (NO) level were pooled. The analysis showed that SBP plus RT had a higher level of NO compared with RT alone (SMD = 0.89, 95% CI [0.65, 1.13], *p* < 0.05; *Q* (2) = 0.15, *p* = 0.93, *I*^2^ = 0, low heterogeneity) (Figure 5(f)).

#### 3.5.5. Complication Rate

**Figure 6:**
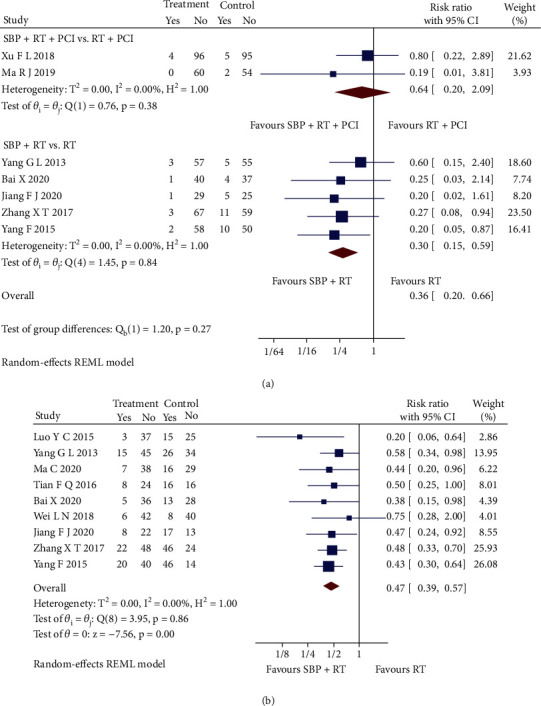
(a) Forest plot of subgroup analysis on the recurrent AMI rate. (b) Forest plot of pooled analysis on the overall complication rate.

Seven RCTs [26, 30, 32, 33, 35, 39, 41] reported recurrent AMI, and nine RCTs [24, 26–28, 30–33, 35] reported complication cases. SBP adjunctive to RT significantly reduced the AMI recurrent rate than RT alone (RR = 0.30, 95% CI [0.15, 0.59], *p* < 0.05; *Q* (4) = 1.45, *p* = 0.84, *I*^2^ = 0, low heterogeneity), but SBP adjunctive to RT plus PCI did not present significant difference (RR = 0.64, 95% CI [0.20, 2.09], *p* > 0.05; *Q* (1) = 0.76, *p* = 0.38, *I*^2^ = 0, low heterogeneity) (Figure 6(a)). As for the overall complication cases, SBP plus RT significantly reduced the overall complication rate than RT alone (RR = 0.47, 95% CI [0.39, 0.57], *p* < 0.05; *Q* (8) = 3.95, *p* = 0.86, *I*^2^ = 0, low heterogeneity), as shown in Figure 6(b).

## 4. Discussion

This review explored the clinical evidence of SBP for AMI in RCTs and antioxidant effects of SBP for AMI in PEs, respectively. In the PEs, we found that SBP enhanced the eNOS activity during oxidative stress and regulated several laboratory indexes involving oxidative damage, including hippuric acid, homocysteine, 5-methylcytosine, PGPC, allantoin indoleacrylic acid, 6-hydroxymelatonin, and thymidine. From the pooled analysis of RCTs, SBP plus RT showed significantly improved clinical efficacy rate, cardiac function, and vascular endothelial function and reduced myocardial enzyme, inflammatory cytokines, blood lipid level, and complication rate. Hence, SBP not only showed the benefits for AMI in RCT but also had antioxidative effects in AMI animal models. Notably, the adjunctive effects of SBP adjunctive to RT plus PCI were diminished in most outcomes except for Hs-CRP and TG levels. It may be attributed to the ceiling effect of PCI for AMI treatment.

**Figure 7:**
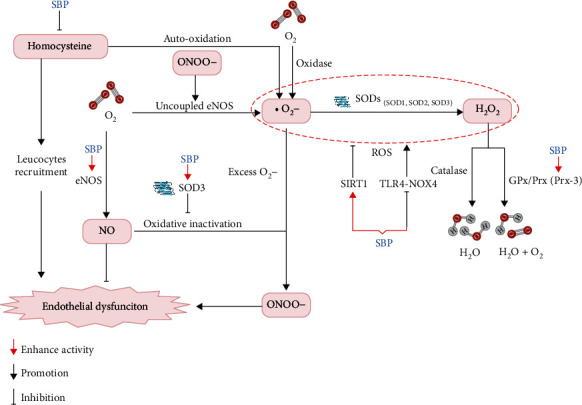
Reactions of the superoxide involving the possible effectiveness of SBP.

Oxidative stress plays a major role in cardiovascular diseases [47–49]. Combined with our preclinical review, SBP removes superoxide anion (O_2_^•−^) through several antioxidative stress mechanisms (Figure 7). SBP protects against endothelial cell injury by activating eNOS activity and promoting nitric oxide (NO) production [50, 51]. Excessive O_2_^•−^ reacts with NO to form peroxynitrite (ONOO^−^) [52–54]. ONOO^−^ in turn oxidizes eNOS to produce O_2_^•−^. Superoxide dismutases (SODs) upregulated by SBP are capable of preserving NO bioactivity [55] and clearing away O_2_^•−^ [56]. Hence, SODs suppress the reaction of O_2_^•−^ and NO from producing ONOO^−^ as well as catalyzing O_2_^•−^ into hydrogen peroxide (H_2_O_2_). H_2_O_2_ finally is detoxified into water and oxygen by glutathione peroxidases (GPx) or peroxiredoxins (Prx) [57]. The study indicates that SBP enhances the activity of peroxiredoxin-3 (Prx-3) to remove H_2_O_2_. Another study reported that reactive oxygen radicals from the autooxidation of homocysteine in plasma probably lead to oxidative damage of endothelial cells [58]. Several active components of SBP have been identified. Cinnamaldehyde decreases the O_2_^•−^ generation through the toll-like receptor 4-NADPH oxidase 4 (TLR4-NOX4) pathway in the lipopolysaccharide-induced cardiac dysfunction [14]. Ginsenoside Rc, one component of SBP, activates a histone deacetylase, sirtuin type 1 (SIRT1), and suppresses ROS [13, 59]. The collecting evidence indicates that SBP has antioxidant effects on AMI. As SBP consists of multiple Chinese medicines, the antioxidant effects could involve multiple active components and multiple mechanisms. A few questions have not been fully addressed; e.g., how many active components are in SBP? What are the mechanisms of these active components suppressing ROS?

This systematic review and meta-analysis included RCTs to evaluate the clinical effects of SBP following the PRISMA guideline. Nevertheless, the overall quality of included RCTs is poor according to the risk of bias assessment, and half of the pooled analysis involving PCI showed high heterogeneity. Compared with RT plus PCI, we found that the SBP adjunctive to RT plus PCI does not significantly affect the AMI recurrence rate and even decreases the LVEF level in one study [40]. Given the inadequate inclusion of RCTs involving PCI, the study cannot confirm the effectiveness of SBP adjunctive to RT plus PCI even though it significantly improves the Hs-CRP and TG levels. Hence, our findings suggest that SBP can be recommended for patients with mild or moderate AMI rather than severe AMI patients requiring PCI treatment. Whether SBP benefits patients with severe AMI needs to be evaluated by rigorous RCTs further.

## 5. Conclusions

SBP protects against oxidative stress in AMI via multiple mechanisms. Clinical evidence indicates that SBP adjunctive to RT improves the clinical efficacy rate, cardiac function, and other clinical indexes of AMI. The current evidence supports the use of SBP for mild and moderate AMI patients.

## Figures and Tables

**Table 1:** Basic information of the included preclinical experiments.

ID	Animal species	Gender	Number of animals	Age	Weight	Intervention	Experimental duration	Experimental type
Luo X P 1999	SD rats (NR)	Male	49	NR	250-300 g	SBP	14 days	Pharmacodynamics
Xiang L 2013	SD rats (NR)	Male	56	NR	200 ± 20 g	SBP, SFSBP, seven components^∗^	15 days	Metabonomics
Liu Q 2017	SD rats (SPF)	Male	81	NR	200 ± 20 g	Ginsenosides in SBP	5 days	Metabonomics
Jiang P 2011	SD rats (NR)	Male	17	NR	200 ± 15 g	SBP	4 days	Metabonomics
Yu F 2021	SD rats (SPF)	Male	6	7 weeks	200-230 g	SBP	15 days	Quantitative proteomics

Note: SD: Sprague-Dawley; SPF: specific pathogen-free; SBP: Shexiang Baoxin Pill; SFSBP: simplified formula of SBP; NR: not reported. ^∗^Including muskone, ginsenoside, ginsenoside, cinnamic acid, cholic acid, bufalin, and borneol.

**Table 2:** Basic information of the included randomized controlled trials.

ID	Sample size (T/C)	Mean age (years)	Diagnostic criteria	Intervention	Comparison	Duration of treatment	Outcomes
Chen ZH 2013	105 (50/55)	T: 58.9 ± 9.3 C: 61.4 ± 10.4	I	SBP plus RT	RT	8 weeks	②④⑤⑥
Luo Y C 2015	80 (40/40)	T: 55.8 ± 19.6 C: 55.8 ± 19.6	II	SBP plus RT	RT	4 weeks	⑧
Ge R L 2015	70 (35/35)	T: 59.9 ± 4.2 C: 60.4 ± 4.1	I	SBP plus RT	RT	8 weeks	②⑤⑥
Yang G L 2013	120 (60/60)	T: 58.2 ± 7.1 C: 56.9 ± 6.8	II	SBP plus RT	RT	24 weeks	②④⑧
Ma C 2020	90 (45/45)	T: 50.5 ± 4.8 C: 50.4 ± 4.3	Compliant with II	SBP plus RT	RT	2 weeks	①②⑤⑦⑧
Tian F Q 2016	64 (32/32)	T: 57.4 ± 5.2 C: 56.5 ± 5.3	II	SBP plus RT	RT	2 weeks	①③④⑧
Wang S S 2016	106 (53/53)	T: 60.5 ± 8.7 C: 61.1 ± 9.1	Compliant with II	SBP plus RT	RT	2 weeks	①②⑤⑦
Bai X 2020	82 (41/41)	T: 56.4 ± 10.5 C: 57.3 ± 10.6	Compliant with II	SBP plus RT	RT	2 weeks	②⑤⑧
Wei L N 2018	96 (48/48)	T: 57.2 ± 8.3 C: 56.9 ± 8.1	Compliant with II	SBP plus RT	RT	2 weeks	①②⑤⑦⑧
Jiang F J 2020	60 (30/30)	T: 40.1 ± 3.4 C: 39.7 ± 4.3	Compliant with II	SBP plus RT	RT	2 weeks	①⑧
Zhang X T 2017	140 (70/70)	T: 63.9 ± 8.6 C: 64.5 ± 8.7	Compliant with II	SBP plus RT	RT	NR	①⑧
Xu J 2017	90 (45/45)	T: 56.2 ± 6.8 C: 55.9 ± 8.6	III	SBP plus RT	RT	12 weeks	②⑥
Yang F 2015	120 (60/60)	T: 64.1 ± 7.9 C: 63.9 ± 8.1	Compliant with II	SBP plus RT	RT	10 weeks	①③④⑧
Feng B 2020	83 (43/40)	T: 57.5 ± 3.3 C: 58.6 ± 3.6	II	SBP plus RT	RT	2 weeks	③④
Huang P D 2016	88 (44/44)	T: 72.2 ± 6.5 C: 72.7 ± 6.1	II	SBP plus RT plus PCI	RT plus PCI	8 weeks	①②③④⑤⑥
Jiang D J 2020	80 (40/40)	T: 55.0 ± 2.4 C: 55.0 ± 2.3	Compliant with II	SBP plus RT plus PCI	RT plus PCI	2 weeks	②⑦
Xu F L 2018	200 (100/100)	T: 59.4 ± 10.1 C: 59.8 ± 10.4	Compliant with II	SBP plus RT plus PCI	RT plus PCI	12 weeks	⑤⑥⑧
Lin G Q 2010	59 (30/29)	T: 60.7 ± 8.2 C: 61.0 ± 7.4	Compliant with II	SBP plus RT plus PCI	RT plus PCI	24 weeks	①②④⑤
Ma R J 2019	116 (60/56)	T: 62.2 ± 8.3 C: 62.0 ± 7.5	Compliant with II	SBP plus RT plus PCI	RT plus PCI	4 weeks	②④⑧

Note: T: treatment group; C: control group; SBP: Shexiang Baoxin Pill; RT: routine treatment (including oxygen inhalation, vascular dilation, anticoagulation, and thrombolysis); PCI: percutaneous coronary intervention; CPGs: clinical practice guidelines; NR: not reported; I: diagnostic criteria of AMI developed by the World Health Organization; II: diagnostic criteria of AMI developed by the Chinese Cardiovascular Society; III: diagnostic criteria of AMI developed by the American Heart Association and American College of Cardiology; ①: clinical efficacy rate; ②: cardiac function; ③: oxidative stress; ④: AMI evaluation index; ⑤: inflammatory factors; ⑥: blood lipid level; ⑦: vascular endothelial function; ⑧: complication rate.

## Supplementary Materials

Supplementary 1 Supplementary Table 1: PRISMA 2009 checklist.

Supplementary 2 Supplementary Table 2: the search strategy of the PubMed database.

Supplementary 3 Supplementary Table 3: risk of bias judgments for preclinical experiments (SYRCLE).

Supplementary 4 Supplementary Table 4: risk of bias judgments for randomized controlled trials (RoB 2.0).
