# Supplementary material for: Shexiang Baoxin Pill for Acute Myocardial Infarction: Clinical Evidence and Molecular Mechanism of Antioxidative Stress
Source: Oxid Med Cell Longev. 2021 Nov 30;2021:7644648. doi: 10.1155/2021/7644648 (PMC8652282; doi:10.1155/2021/7644648)
Supplement: Supplementary 2 — Supplementary Table 2: the search strategy of the PubMed database. [file 7644648.f2.docx]

**Supplementary Table 2. The search strategy of the PubMed database**

| **Number** | **Keywords of the Research** |
| --- | --- |
| **#1** | Acute myocardial infarction[mh] |
| **#2** | Myocardial infarction*[tiab] OR Acute myocardial infarction[tiab] OR Coronary syndrome[tiab] |
| **#3** | OR/#1-#2 |
| **#4** | Shexiang Baoxin Pill[mh] OR Heart-Protecting Musk Pill[mh] |
| **#5** | Shexiang Baoxin*[tiab] OR Shexiang Baoxin Pill[tiab] OR Shexiangbaoxin[tiab] OR SBP[tiab] OR SXBX[tiab] [tiab] OR Heart-Protecting Musk*[tiab] OR Heart-Protecting Musk Pill[tiab] OR HPM[tiab] |
| **#6** | OR/#4-#5 |
| **#7** | #3 AND #6 |

Note: mh: MeSH; tiab: tittle/abstract
