# Supplementary material for: Shexiang Baoxin Pill for Acute Myocardial Infarction: Clinical Evidence and Molecular Mechanism of Antioxidative Stress
Source: Oxid Med Cell Longev. 2021 Nov 30;2021:7644648. doi: 10.1155/2021/7644648 (PMC8652282; doi:10.1155/2021/7644648)
Supplement: Supplementary 3 — Supplementary Table 3: risk of bias judgments for preclinical experiments (SYRCLE). [file 7644648.f3.docx]

**Supplementary Table 3. Risk of bias judgments for preclinical experiments (SYRCLE)**

| **ID** | **Sequence generation** | **Baseline characteristics** | **Allocation concealment** | **Random housing** | **Blinding of performance** | **Random outcome assessment** | **Blinding of detection** | **Incomplete outcome data** | **Selective outcome reporting** | **Other sources of bias** |
| --- | --- | --- | --- | --- | --- | --- | --- | --- | --- | --- |
| Luo X P 1999 | Some concerns | Some concerns | Some concerns | Some concerns | Some concerns | Some concerns | Low | Low | Low | Some concerns |
| Xiang L 2013 | Low | Some concerns | Low | Some concerns | Some concerns | Low | Low | Low | Low | Low |
| Liu Q 2017 | Some concerns | Low | Some concerns | Some concerns | Some concerns | Some concerns | Low | Low | Low | Some concerns |
| Jiang P 2011 | Some concerns | Low | Some concerns | Some concerns | Some concerns | Some concerns | Low | Low | Low | Some concerns |
| Yu F 2021 | Low | Some concerns | Low | Some concerns | Some concerns | Low | Low | Low | Low | Low |
