# Supplementary material for: Shexiang Baoxin Pill for Acute Myocardial Infarction: Clinical Evidence and Molecular Mechanism of Antioxidative Stress
Source: Oxid Med Cell Longev. 2021 Nov 30;2021:7644648. doi: 10.1155/2021/7644648 (PMC8652282; doi:10.1155/2021/7644648)
Supplement: Supplementary 4 — Supplementary Table 4: risk of bias judgments for randomized controlled trials (RoB 2.0). [file 7644648.f4.docx]

**Supplementary Table 4. Risk of bias judgments for randomized controlled trials (RoB 2.0)**

| **ID** | **Bias arising from the randomization process** | **Bias due to deviations from intended intervention** | **Bias due to missing outcome data** | **Bias in measurement of the outcome** | **Bias in selection of the reported result** | **Overall** |
| --- | --- | --- | --- | --- | --- | --- |
| Chen Z H 2013 | Some concerns | Low | Some concerns | Low | Some concerns | Some concerns |
| Luo Y C 2015 | Some concerns | Low | Some concerns | Low | Some concerns | Some concerns |
| Ge R L 2015 | Some concerns | Low | Some concerns | Low | Some concerns | Some concerns |
| Yang G L 2013 | Low | Low | Some concerns | Low | Some concerns | Some concerns |
| Ma C 2020 | Low | Low | Low | Low | Low | Low |
| Tian F Q 2016 | Low | Low | Some concerns | Low | Low | Low |
| Wang S S 2016 | Low | Low | Low | Low | Some concerns | Low |
| Bai X 2020 | Low | Low | Some concerns | Some concerns | Some concerns | Some concerns |
| Wei L N 2018 | Low | Low | Some concerns | Some concerns | Some concerns | Some concerns |
| Jiang F J 2020 | Low | Low | Some concerns | Some concerns | Some concerns | Some concerns |
| Zhang X T 2017 | Low | Low | Some concerns | Low | Some concerns | Some concerns |
| Xu J 2017 | Some concerns | Low | Some concerns | Some concerns | High | High |
| Yang F 2015 | Low | Low | Some concerns | Low | Some concerns | Some concerns |
| Feng B 2020 | Low | Low | High | High | Some concerns | High |
| Huang P D 2016 | Low | Low | Low | Low | Low | Low |
| Jiang D J 2020 | Low | Low | Some concerns | Low | Some concerns | Some concerns |
| Xu F L 2018 | Low | Low | Some concerns | Low | Some concerns | Some concerns |
| Lin G Q 2010 | Some concerns | Low | Low | Low | Some concerns | Some concerns |
| Ma R J 2019 | Low | Low | Some concerns | Low | Some concerns | Some concerns |
